# Food Safety Issues in the Oltrepò Pavese Area: A SERS Sensing Perspective

**DOI:** 10.3390/s23229015

**Published:** 2023-11-07

**Authors:** Benedetta Albini, Pietro Galinetto, Serena Schiavi, Enrico Giulotto

**Affiliations:** 1 Dipartimento di Fisica, Università di Pavia, Via Bassi 6, 27100 Pavia, Italy; benedetta.albini@unipv.it (B.A.); pietro.galinetto@unipv.it (P.G.); 2 Dipartimento di Chimica, Università di Pavia, Via Taramelli 12, 27100 Pavia, Italy; serena.schiavi01@universitadipavia.it

**Keywords:** food safety, surface-enhanced raman scattering, emerging pollutants

## Abstract

Handly and easy-to-use optical instrumentation is very important for food safety monitoring, as it provides the possibility to assess law and health compliances at every stage of the food chain. In particular, the Surface-enhanced Raman Scattering (SERS) method appears highly promising because the intrinsic drawback of Raman spectroscopy, i.e., the natural weakness of the effect and, in turn, of the signal, is overcome thanks to the peculiar interaction between laser light and plasmonic excitations at the SERS substrate. This fact paved the way for the widespread use of SERS sensing not only for food safety but also for biomedicine, pharmaceutical process analysis, forensic science, cultural heritage and more. However, the current technological maturity of the SERS technique does not find a counterpart in the recognition of SERS as a routine method in compliance protocols. This is mainly due to the very scattered landscape of SERS substrates designed and tailored specifically for the targeted analyte. In fact, a very large variety of SERS substrates were proposed for molecular sensing in different environments and matrices. This review presents the advantages and perspectives of SERS sensing in food safety. The focus of the survey is limited to specific analytes of interest for producers, consumers and stakeholders in Oltrepò Pavese, a definite regional area that is located within the district of Pavia in the northern part of Italy. Our attention has been addressed to (i) glyphosate in rice fields, (ii) histamine in a world-famous local product (wine), (iii) tetracycline, an antibiotic often detected in waste sludges that can be dangerous, for instance in maize crops and (iv) Sudan dyes—used as adulterants—in the production of saffron and other spices, which represent niche crops for Oltrepò. The review aims to highlight the SERS performance for each analyte, with a discussion of the different methods used to prepare SERS substrates and the different reported limits of detection.

## 1. Introduction

The present review is focused on a critical analysis of the use of Raman and Surface Enhanced Raman Spectroscopy (SERS) for food safety. Food safety issues can be generally defined as the presence of unexpected or unidentified (on the product label or on the traceability certificates) physical, chemical, or biological contaminants. These issues have a twice as important impact. Primarily, they represent a serious public health problem with relevant societal fall-out. Secondly, they constitute a serious and recurrent economical problem as they are a heavy threat for producers, industries and public entities operating in the global food market [1].

Food contamination can be categorized as biological, physical, and chemical, depending on its nature and origin. Typical food hazards are chemicals, bacteria, viruses, parasites, prions. Concerning chemicals, we refer to naturally occurring toxins, Persistent Organic Pollutants (POPs), heavy metals and other chemical hazards as, for instance, radioactive nucleotides, food allergens, residues of drugs and other contaminants incorporated in the food during the production process [2]. Chemical contamination can lead to acute poisoning or long-term diseases, such as cancer. Many foodborne diseases may lead to long-lasting disability and death.

Foodborne illnesses are usually infectious or toxic in nature and caused by bacteria, viruses, parasites or chemical substances entering the body through contaminated food [3]. Food contamination describes the event at which a foreign material or substance that can induce foodborne illnesses is introduced into the food material. These events can occur at any step in the food supply chain from producer to consumer in the course of production, processing, distribution, retailing and consumption. Indeed, contamination of raw materials can occur from the soil, sewage, live animals, external surface, and the internal organs of meat animals. Other sources of contamination include water, air, dust, equipment and employees. Heavy metal contamination in food occurs mainly through pollution of water and soil [4].

According to the European Regulation (EC) No 852/2004 [5] (with subsequent amendments (EC) No 1019/2008, Regulation (EU) No 579/2014 and Regulation (EU) 2021/382) the aim of the legislator is to ensure and verify the food composition and quality with the so-called farm to fork approach, i.e., from farms to processing plants and retailers to the final consumer. The key principle is that every subject working in the food chain (and business) must ensure that food is processed free of contamination from foodborne hazards, at every stage of the production process. This must be achieved through good hygiene practices and procedures based on the hazard analysis and critical control points (HACCP) principles [6]. Among the procedures based on the HACCP principles, the effective monitoring of food contamination is a key element to ensure the quality of the whole food chain. Thus, it is easy to understand why many different advanced techniques have been proposed and used for monitoring in the food chain. The ensemble of advanced analytical techniques and methods satisfactorily employed for food safety is extremely wide and it strongly depends on the contaminants to be detected, the raw materials or matrices where the contaminants may be expected, the food chain frame where the technique must be used and the rules and regulations of HACCP strategy that must be satisfied. For instance, traditional analytical methods for the detection of food additives mainly include chromatography [7], such as liquid chromatography, gas chromatography and mass/liquid chromatography. The use of all these techniques for food safety purposes is well established. Nevertheless, all these methods typically require large-scale laboratory instruments and a complex chemical preparation of the samples, thus hindering a real-time and on-site approach.

Emerging analytical methods for food safety exploit spectroscopic, electrochemical, electrical, and magnetic detection [8]. In some cases, the real-time and on field approach is guaranteed by the physics of the effect used to detect specific chemical or biological targets. This is the case of Raman spectroscopy and related techniques (microRaman, Resonant Raman Spectroscopy (RRS) [9], SERS [10,11] and Spatially Offset Raman Spectroscopy (SORS) [12]). Raman spectroscopy presents a number of advantages for food safety assessment. As explained in Section 2, the technique is based on the study of the inelastic scattering from the matter when irradiated with a laser light usually in the visible or near-infrared range. The Raman technique can provide a molecular spectral fingerprint, it is usually very rapid and non-destructive and easy-to-use for non-expert professionals as well. In addition, it can operate in water matrices since water is a weak Raman scatterer. It is important to note that the main limit of Raman spectroscopy, i.e., the intrinsic weakness of inelastic light scattering, can be overcome by using the SERS approach or in general those approaches exploiting the field and chemical enhancement effects able to boost the Raman signal. Finally, Raman spectroscopy, thanks to optical fiber transduction and modern integrated optical components, is completely transferable to field instrumentation.

Raman and SERS spectroscopies for food safety have been used for all the possible food hazards described above. Many examples can be found in the literature [13,14] and some contributions come from our research group [15,16,17,18,19]. To illustrate the state-of-the-art with the proper depth and accuracy, we do not attempt an excursus on all the different uses of Raman spectroscopy in food safety, but we restrict our focus to some specific sources of food contamination which can take place in three different food chain steps: (i) cultivation of raw materials, (ii) food preparation and (iii) food conservation. In order to better define our targets, we further restrict our interests using territorial and economic filters, taking into account the territory in which the University of Pavia is located. For the first two points, we have considered two of the flagships of the Oltrepò Pavese area, namely crops, like rice and maize, and grapes. According to 2020 production figures, about 7 million quintals of rice and maize were produced and 1 million tons of grapes were destined for wine production [20]. For crops we have considered contamination sources, the irrigation water and sludge and sewage used as fertilizers. For grapes, we have considered the natural presence of contaminant molecules as a result of the bacterial activity in the food preparation procedure, in this case the wine production. For the third point, we have considered saffron, a very precious spice obtained from the stigmas of the Crocus Sativus flower. The cultivation of saffron in Italy is increasing, with a total production of about 500–600 kg per year. Production is also increasing in Oltrepò Pavese where approximately 1% of the whole Italian saffron is produced. The adulteration issue is clearly related to the high price of saffron (20,000–25,000 EUR/kg) [21].

**Scheme 1:**
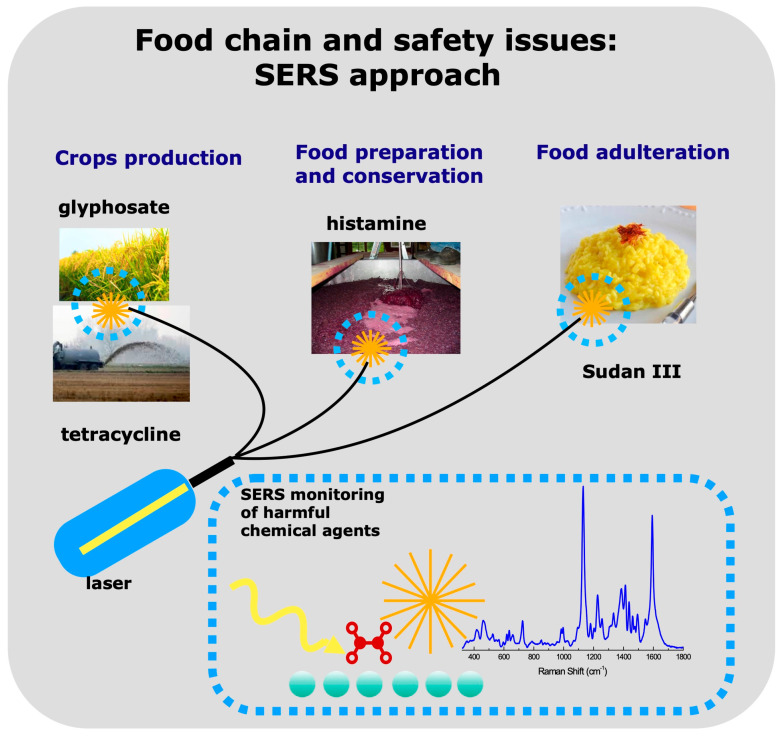
Conceptual scheme of the SERS approach in food safety monitoring for the detection of harmful chemical agents in different food chain steps.

Based on the above-described criteria, this review (see Scheme 1 for a graphical abstract) presents the state of the art of SERS detection of:

- glyphosate, a pesticide widely used in rice cultivations
- tetracycline in cultivation soils after sludge dispersion
- histamine in wine
- Sudan in saffron and other spices.

## 2. Raman Scattering and SERS

When a laser beam of monochromatic wavelength λ_0_ impinges on an ensemble of molecules, a fraction of the light intensity is scattered in all directions, mostly elastically, i.e., with no change in wavelength. Only a tiny fraction of the scattering events (typically around 10^−7^) is inelastic, i.e., the scattered and incident photon wavelengths are different. The energy shift ΔE = hcΔ(1/λ) is known as the Raman shift [see for instance [22] and references therein]. A negative (Stokes) shift corresponds to the excitation of a vibrational mode of the molecule of frequency Δν = cΔ(1/λ), whereas a positive (anti-Stokes) shift involves the de-excitation of a vibrational mode. Conventionally, however, Stokes line intensities are plotted vs. positive energy (or wavenumber) shift semiaxis. At ordinary temperatures, the anti-Stokes lines are weaker than the corresponding Stokes lines due to the low population of the excited states.

Different molecules possess vibrational modes with different characteristic frequencies, so that the Raman spectrum of a molecule can be regarded as a fingerprint of the molecule itself. To be Raman active, a vibrational mode must produce a modulation in some component of the polarizability tensor, with frequency Δν. Typical strong Raman scatterers are moieties with distributed electron clouds, such as C=C bonds, whereas highly polar moieties, such as the O-H bond, are usually weak Raman scatterers.

Laser sources in the visible or near infrared range are normally used. Interference of Raman scattering with fluorescence is a frequently encountered inconvenience. This can be disruptive as fluorescence intensity is often much larger than the weak Raman signal. The use of laser light with relatively long wavelengths may avoid or minimize the problem.

In many applications, Raman spectroscopy is recognized for its potential. Provided that laser power is low enough not to damage the sample, it is a non-destructive technique, and usually requires minimal sample preparation. Unlike infrared vibrational spectroscopy, it can be used on samples in aqueous media. Many modern Raman spectrometers are coupled with a microscope objective, so that lateral spatial resolutions of 1 μm or better can be achieved. Rapid on-site detection, a key element in several applications, can be ensured by handheld instruments. Indeed, the development of compact diode lasers with a range of excitation wavelengths and improved detector technologies with methods for fluorescence rejection opened new routes for applications [23]. These advances have made Raman spectroscopy an analysis technique of practical use in the laboratory as well as in field research [24]. An exhaustive overview on the progress of portable Raman spectroscopic devices from transportable Raman spectrometers to portable, handheld, and, eventually, palm-sized Raman spectrometers can be found in [25].

In Surface-enhanced Raman Scattering, the signal enhancement produced by the proximity (adsorption) of sample molecules to a nanostructured metal surface, or metal nano-objects, is used to increase the sensitivity of the technique. The metal is usually gold or silver. Large enhancement factors can be achieved if the laser wavelength is close to a localized surface plasmon resonance of the metal particle. There may be nanoregions (hotspots), such as tips or particle-particle gaps where the enhancement is very strong. In favorable cases the enhancement factor can be as large as 10^10^ to 10^11^, and the technique can detect single molecules. A drawback of the SERS technique resides in the difficulty to achieve high repeatability/reproducibility of signal intensity as well as position and width of spectral lines. Poor selectivity can also be an issue.

SERS-based sensors can be classified according to three main categories [26]. The first category includes methods in which the Raman signal from the analyte is directly detected. The analyte can be either adsorbed to the nano-structured metal substrate or bound to a functionalized substrate. The use of unfunctionalized structures as sensors suffers from lack of selectivity. Functionalized surface schemes can highly improve selectivity, but they may be affected by the Raman signal from the functionalization layer overpowering the signal from the analyte. The second approach relies on SERS-tagged nanoparticles. The Raman signal of interest originates from the SERS tags (or Raman reporters). Selectivity is ensured by analyte-targeting ligands attached to the nanoparticles. In the third strategy the signal from the SERS tags is recorded, and a specific change in the Raman spectrum resulting from the interaction with the analyte is detected. (a) The interaction may result in an absorption band close to the exciting wavelength and, in turn, to resonance Raman enhancement. (b) The analyte–probe interaction may bring the probe in closer contact with the substrate, resulting in enhanced Raman signal. (c) Chemical/conformational changes in the probe molecules may be caused by the analyte. This will result in detectable modifications of the Raman spectrum.

## 3. SERS Detection of Glyphosate

**Figure 1:**
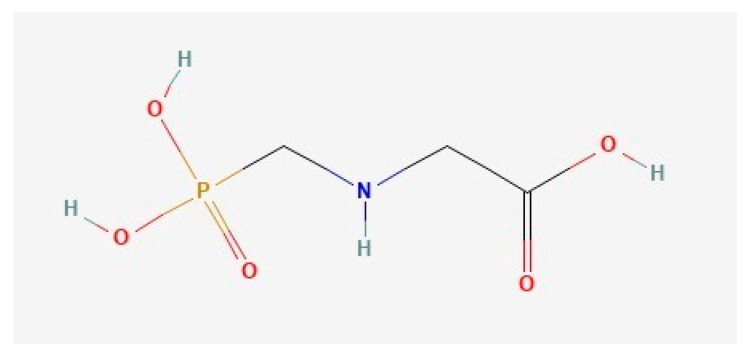
Chemical structure of Gly (retrieved from [27]).

Glyphosate (Gly) (C_3_H_8_NO_5_P) is a phosphonic acid whose proper chemical name is N-(Phosphonomethyl)glycine [27] (see Figure 1 for the chemical structure). This chemical is the most diffused component in plant protection products (PPPs), and it is highly employed as a pesticide in agriculture and horticulture [28].

The widespread use of this pesticide is due to the fact that, at the time of its introduction on the market in the 1970s, it was one of the least harmful pesticides because it only affects weeds, in view of the introduction of genetically modified Gly-resistant crops in cultivated fields. In addition, Gly began to be widely used even in urban environment to keep roads and railways free from weeds [29].

There is an open controversy on toxicity of Gly to human health. In particular, in 2015 the International Agency of Research on Cancer (IARC) classified glyphosate as *probably carcinogenic*, in view of evidence on real cases in humans and in laboratory animals, as well as important evidence of genotoxicity [30]. However, its potential toxicity is related to how it is used, direct contact, and time of exposure. For example, in Brazil, glyphosate is spread by airplanes, thus involving the urban areas next to the cultivated fields, consequently causing direct contact of the population with the pesticide. On the other hand, in Europe, more strict rules have been set on its use. Since 2016 in Italy, where the glyphosate issue is primarily related to rice growth [31], the use of this herbicide is only allowed in agriculture and forbidden in urban areas and green areas frequented by people and children, like parks, playgrounds and gardens. Moreover, even in agriculture, its use is not allowed in the pre-harvest period [32]. Despite this rigid protocol, since 2022, there is an alert in the district of Pavia for the high level of glyphosate and its metabolite AMPA (Aminomethylphosphonic acid) in surface waters, in particular irrigation channels, as well as groundwater [33,34].

Currently, the use of glyphosate is allowed in Europe until December 2023. The European Food Safety Authority (EFSA) *“does not identify any critical areas of concern that would prevent the renewal of the approval of glyphosate”*, as reported in the summary of the discussion between the Commission services and the Member States in the Standing Committee on Plants, Animals, Food and Feed (PAFF Committee) [35]. However, in its peer review [36], EFSA underlined that the renewal is not hindered provided that some mitigation measures are set to address several issues that could not be finalized and other outstanding issues due to lack of data. In order to finalize the decision on glyphosate’s renewal within the actual expiring date, a further PAFF meeting will be held in September 2023 to define a draft regulation [28]. Since the potential toxicity of glyphosate is strictly related to its use, as already stressed, and in view of its potential carcinogenic action, a careful monitoring of its presence in crops and environment is mandatory, with the assessment that its values do not overcome the maximum residual limits (MRLs) imposed by EFSA [37].

As already pointed out, Raman spectroscopy could be a valid candidate for in situ monitoring. However, since the new MRLs, reviewed in 2019, are between 0.05 and 1 ppm, only Surface Enhanced Raman Spectroscopy may be expected to achieve the requested sensitivity.

**Figure 2:**
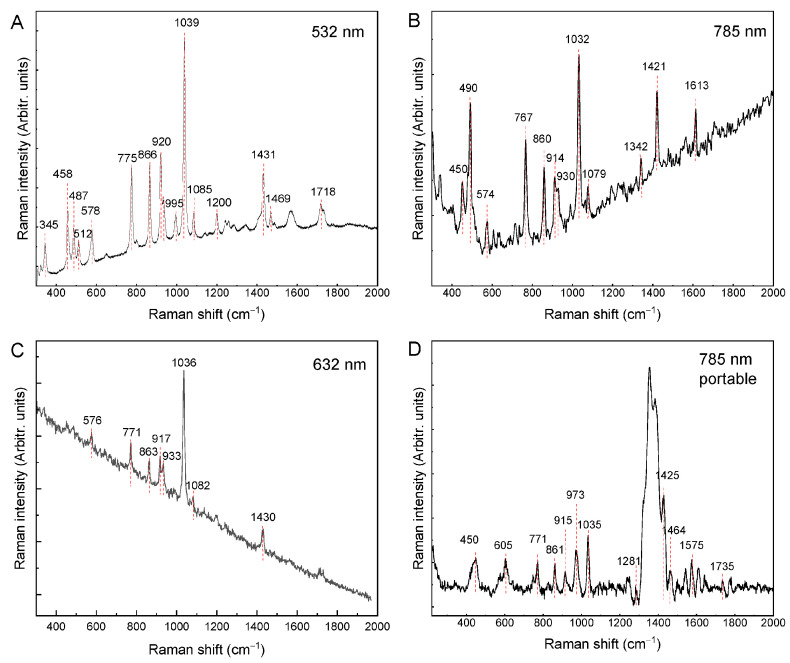
Room temperature Raman spectra of Gly powder at different exciting laser wavelengths ((**A**) 532 nm, (**B**) 785 nm, (**C**) 632 nm and (**D**) 785 nm (portable instrumentation)), retrieved from [38].

Glyphosate is characterized by an intense Raman activity between 300 and 1800 cm^−1^, as shown in Figure 2, where the Raman signal is reported at different excitation wavelengths [38]. The most prominent mode, in spectra collected with benchtop Raman instrumentation, is centered at 1032 ÷ 1039 cm^−1^ and it was tentatively assigned to the symmetric stretching of PO_2_ [39] or to C-N stretching, CNCC skeleton vibration and HOPO_2_ as POO antisymmetric stretching [40].

The cited article [38] and other research works, like for instance [39,41,42], deal with an important aspect in view of SERS application for Gly detection, namely the changes occurring between the Raman and SERS spectra. Indeed, the characterization of the interaction between the analyte and the metallic surface is important to optimize the performance of the SERS sensor itself and, therefore, to improve its sensitivity for routine sampling and in situ detection. A significant difference between the Raman spectrum of Gly powder and its SERS spectrum is commonly observed, in terms of modifications in the modes’ relative intensity ratio as well as in their positions. There is common agreement that the adsorption of glyphosate on the metallic surface generally occurs through the phosphate groups, thanks to the detailed analysis of such changes.

**Figure 3:**
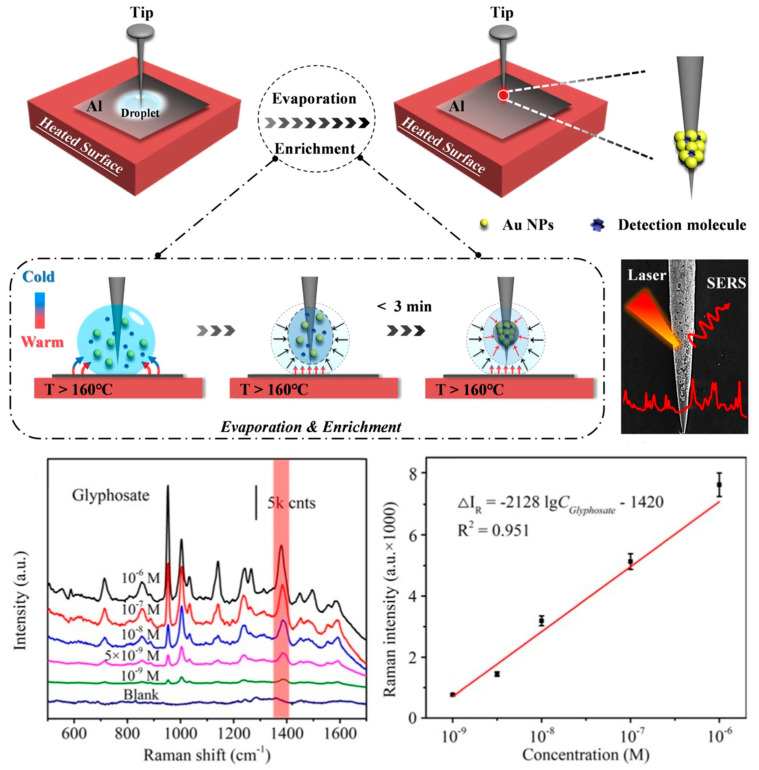
Schematic illustration of needle tip enrichment approach with Au nanoparticles for SERS sensing (**top**). Spectral intensities and linear regression curve as a result of the application of the method to Gly detection (**bottom**). Retrieved from [53].

An increasingly body of literature is available on the use of SERS for glyphosate detection both as a single detection technique as well as part of a combined approach [43,44,45]. We point out that the majority of research papers [39,41,42,44,45,46,47,48,49,50,51] illustrates studies on Gly detection by using Ag nanoparticles as an enhancing active medium, and bench-top Raman instrumentation to perform the measurement, reaching Limits Of Detection (LODs) generally below the MRLs imposed by the European Union (EU). In contrast, refs [52,53] present two different research works exploiting, respectively, Au-Ag nanochains for a novel anti-interference, indirect detection, reaching a LOD of 5 μg/L (5 ppb) and Au nanoparticles in a needle tip enrichment approach with an estimated LOD of 7.5 nmol/L (≈1.3 ppb) (see Figure 3 for an illustration of the sensing mechanism and related SERS results). In both these studies, SERS measurements were performed by means of portable Raman instruments. In particular, in [53] the instrumental specifications are follows: iRaman, BWTEK, portable spectrometer equipped with a 40× objective lens and a 785 nm (30 mW) laser source.

## 4. SERS Detection of Tetracycline

The extensive use of antibiotics is an urgent issue nowadays. With an average consumption in Europe of 16.4 daily doses per 1000 inhabitants, to which the consumption for animal healthcare should be added, their presence in the environment is producing high levels of contamination [54]. Indeed, the main problem is that human and animal bodies cannot completely metabolize antibiotics. Therefore, a certain quantity is excreted and contaminates urban wastewater and water treatment plants. With a chain effect, these contaminants affect watercourses, lakes, sea, and soil, if it is fertilized by sewage sludge.

The antimicrobial resistance, namely the ability of bacteria to resist to the antibiotic effect, is increasing, which also increases the hazard caused by antibiotic pollution [55]. In this regard, a research carried out at the Department of Clinical-Surgical, Diagnostic and Pediatric Sciences of the University of Pavia [56] has demonstrated the presence of different antibiotic resistant bacteria, like for instance Escherichia coli [57], in surface and groundwater of urban and suburban areas of Pavia.

Tetracycline (TET) is a broad-spectrum antibiotic, widely employed for both human and animal care in view of its high performance combined with its low cost. Its action is potent against different Gram-positive and Gram-negative bacteria, protozoan parasites, including atypical organisms such as mycoplasma, chlamydia, and rickettsia. A detailed analysis of the tetracycline’s issue in the aquatic environment is presented in [58]. Because of the widespread presence of tetracycline’s residues in the environment and especially in the food chain, a careful monitoring is needed to control that its concentration is below the MRLs imposed by the EU, namely 0.1 ppm in muscle and milk, 0.2, 0.3, 0.6 ppm in eggs, liver and kidney, respectively [59].

**Figure 4:**
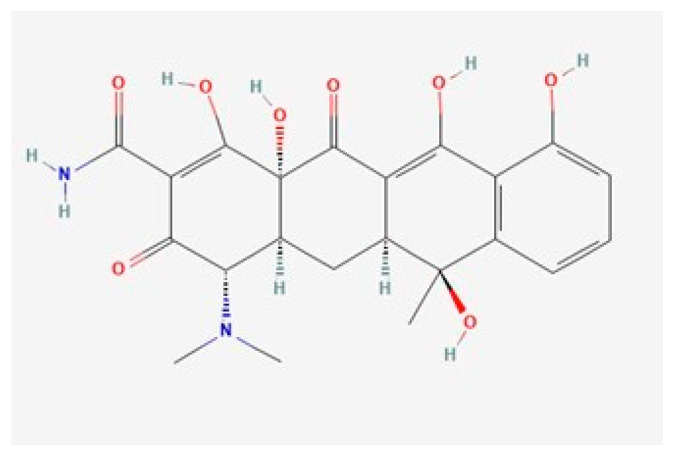
Molecular structure of Tetracycline retrieved from [60].

Figure 4 reports TET’s molecular structure (C_22_H_24_N_2_O_8_) [60].

**Figure 5:**
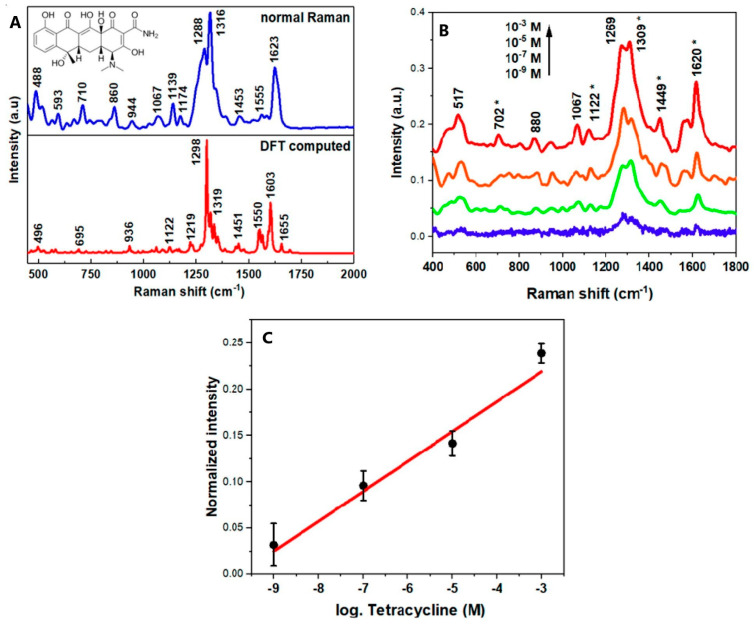
(**A**) Raman spectrum of 0.1 mol/L aqueous TET solution (upper panel) collected by using a 785 nm laser source and (lower panel) computed by DFT calculations. (**B**) SERS spectra of tetracycline in milk at different concentration levels (the asterisks (*) indicate the TET modes) and (**C**) corresponding values of SERS intensity at 1620 cm^−1^ as a function of TET concentration. Retrieved with permission from [61]. Copyright 2020 American Chemical Society.

Its Raman spectrum (see Figure 5A) is characterized by an intense activity between 450 and 1750 cm^−1^. The most intense modes fall around 1300 cm^−1^ and 1620 cm^−1^. A detailed attribution of TET’s modes can be found in [61,62]. In particular, in [62], a comparison between the Raman signatures of fully protonated tetracycline in aqueous environment and those of tetracycline hexahydrate in the crystalline state is presented.

The use of tetracycline for animal health care can also result in the presence of TET residuals in livestock products, like, for instance, milk and eggs [63]. Thus, it is mandatory to verify that the presence of this antibiotic does not exceed the MRLs imposed by the EU. In this regard, some research works have dealt with the analysis of milk by means of surface-enhanced Raman spectroscopy [61,64,65,66]. As an explanatory case, the results obtained by Muhammad et al. [61] are reported in Figure 5B,C. A LOD of 0.4 ppb is claimed. In [64], a low-cost SERS sensor based on Ag nanoparticles on a cardboard packaging substrate is used to detect TET spiked in milk with concentration ranging from 0.01 to 1000 ppm. The analysis of the collected Raman spectra on dried drops was performed through a Principal Component Analysis (PCA) approach [67], demonstrating a sensitivity of 0.1 ppm.

S. Dhakal et al. [65] performed a SERS investigation making use, also in this case, of Ag nanoparticles, but in colloidal form. The measurements were performed on both water- and milk-tetracycline solutions on spiked wet drops by means of a portable Raman spectrometer, Raman Explorer 785, Headwall Photonics, Fitchburg, MA, USA, connected through a 100 μm slit to one end of a bifurcated optical fiber. The other end of the fiber was connected to a 785 nm laser module (I0785MM0500MF, Innovative Photonics Solutions, Monmouth Junction, NJ, USA) to deliver the light source for sample excitation. A LOD of 0.01 ppm was demonstrated.

More elaborated SERS substrates based on Ag nanostructures are presented in refs [61,66]. In particular, the first work presents a transparent substrate composed of highly ordered Ag nanoparticles (NPs) arrays fabricated with a protocol based on the anodic aluminum oxide template-assisted electrochemical deposition plus acid etching and by finally sputtering the Ag NPs. This approach allowed to reach a LOD of 1 × 10^−9^ mol/L (≈0.44 ppb). The second work uses a PDMS substrate. The TET-spiked milk was injected in the PDMS substrate enriched by Ag nanoparticles, then sucked out after 6 min and then the SERS measurements were performed, reaching a detection limit of 0.28 ppb.

Another recent article [68] reports on a SERS substrate of Ag nanodisks deposited on common filter paper able to detect TET down to 10^−9^ mol/L (≈0.44 ppb) in water solution.

## 5. SERS Detection of Histamine

**Figure 6:**
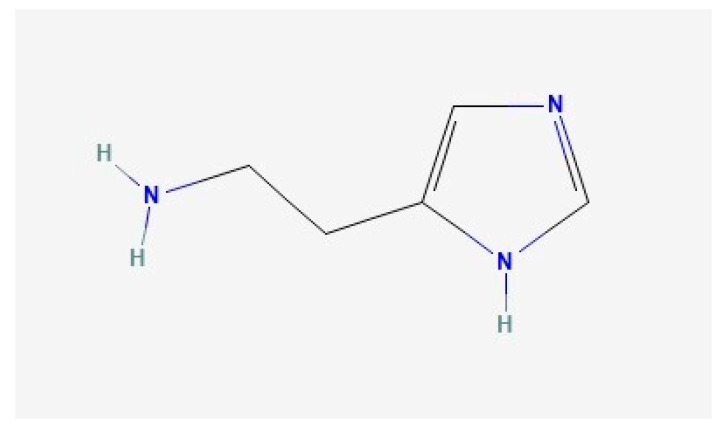
2D molecular structure of histamine, retrieved from [70].

Histamine (His) is a biogenic amine composed by an imidazole ring attached to an ethylamine chain (C_5_H_9_N_3_) (see Figure 6 for the molecular structure). This amine is the product of the enzymatic decarboxylation of histidine, which is an amino acid naturally present in many tissues in the human body [69]. The endogenous histamine is mainly stored in masts cells and basophils and its release in the body is promoted by different stimulations.

Although His is involved in many physiological processes as neurotransmitter [71], it is mainly known for the diffused intolerance associated with it. Some individuals cannot properly metabolize exogenous histamine. This may cause allergy-like symptoms, such as rush, general effects on the gastrointestinal tract, low blood pressure and headache [72,73]. Indeed, this amine is naturally present in the animals’ tissues, plants, and food like foodstuffs that have undergone the fermentation process, as wine. Indeed, the primary source of wine intolerance lies in the presence of histamine’s high concentration [74]. The origin of the occurrence of high or low levels of His in wine is still controversial, although studies have demonstrated that the variation in His level seems independent of the grape variety [75,76], while a possible dependence on the production process could be assumed, in particular by selecting a proper bacterial strain [77].

No limit is currently set by the EU on His content in wine, although the International Organization of Vine and Wine advises the winemakers not to exceed 12 mg/L, roughly 12 ppm [77].

**Figure 7:**
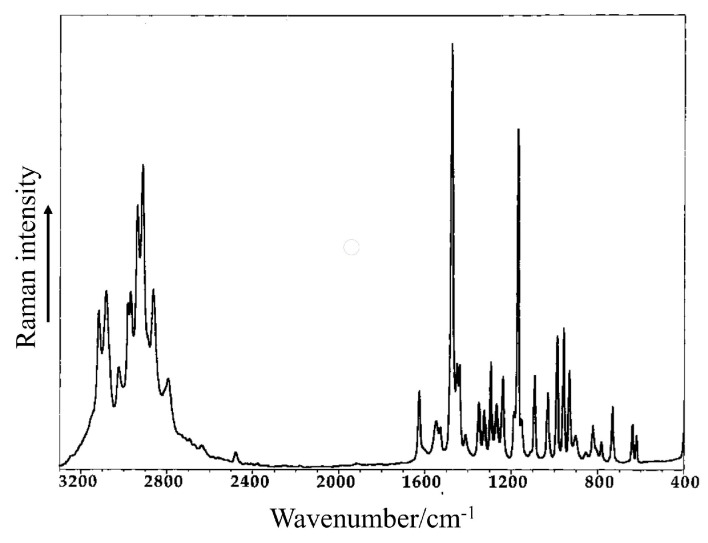
Raman spectrum of natural histamine dihydrochloride microcrystalline powder. The 1064 nm radiation from a Nd: YAG laser was used. Retrieved with permission from [78].

Figure 7 reports the Raman spectra of histamine dication in solid powder. The high wavenumber region is dominated by the activity related to C-H and N-H stretching modes. An intense Raman activity is also registered between 600 and 1700 cm^−1^. A detailed assignment of solid His modes as well as of histamine diluted in normal and heavy water can be found in [78,79] for dihydrochloride and free-base amine, respectively.

A fairly wide literature is available on the detection of histamine in food by means of SERS. According to the topic of the present review, we restrict our focus on His detection in wine by SERS. However, we also consider works where detection takes place in liquids or simply treat the problem of histamine detection from a general point of view.

Ref. [80] deals with SERS detection of histamine in wine. An AgNPs-cellulose hybrid substrate is proposed as an easy approach to detect His in liquid. A LOD of pmol/L (about 1 × 10^−4^ ppb) for histamine in water is claimed. The measurements were performed by simply dropping the liquid sample on the substrate and using a bench-top Raman system with a 532 nm laser source. As a proof of concept, this cellulose-based device was also tested on real samples: a commercial and a more expensive white wine, stored in tetra pack and glass, respectively. The sensitivity of the sensor was confirmed by means of high-performance liquid chromatography (HPLC) measurements.

A novel approach for both extracting and sensing histamine in food is proposed in [81] by exploiting the molecular imprinted polymers (MIPs) strategy to improve the selectivity of the substrate itself. Upconversion particles (UCNPs) were covered by a layer of MIP doped with AgNPs. Thus, the UCNPs@MIPs–AgNPs sensor simultaneously enhanced the Raman signal and quenched the fluorescence response. The SERS measurements on His were performed by depositing the UCNPs@MIPs–AgNPs pellet obtained after centrifugation of the colloidal suspension onto a gold-coated microarray chip. Before mixing, the pristine UCNPs@MIPs–AgNPs colloids were spiked with different concentrations of His. The SERS spectra were characterized by the four more intense His modes at 1268 cm^−1^, 1331 cm^−1^, 1572 cm^−1^ and 1427 cm^−1^. Among them, the one at 1331 cm^−1^, ascribed together with the ones at 1268 cm^−1^ and 1572 cm^−1^ to the stretching and breathing of the imidazole ring, is reported to be the most dose-sensitive, which allowed to obtain a good calibration curve. A LOD of 0.04 ppm is reported. The reliability of the proposed sensors was tested in real food samples, namely red wine, rice wine and canned tuna spiked with three different known quantities of histamine (5, 15 and 30 mg/L). The procedure showed a good accordance between the results obtained with UCNPs@MIPs–AgNPs sensors and with HPLC tests.

**Figure 8:**
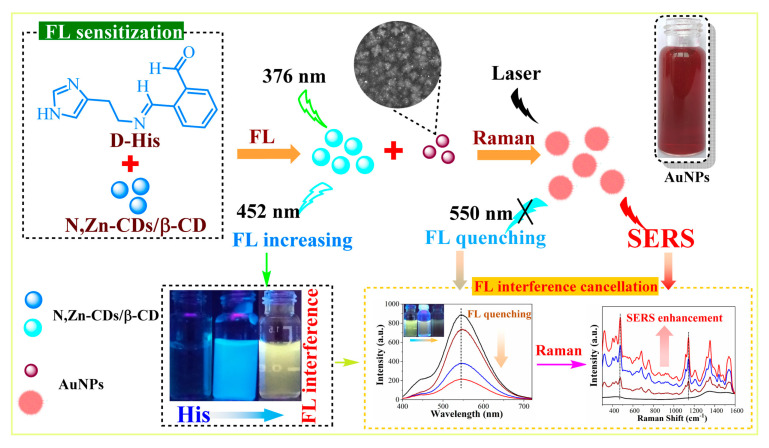
Schematic illustration of the sensing process proposed by Yang et al. Retrieved from [82].

In Ref. [82], the authors describe a sensor based on Au NPs combined with carbon dots. They demonstrate that after derivatization of His (D-His), a key-passage for His detection, the presence of β-cyclodextrin (β-CD) decorated N,Zn co-doped carbon dots was decisive in the detection of a clear D-His SERS signal. The sensing strategy is illustrated in Figure 8. The LOD attainable with the proposed substrate was about 10 μmol/L (≈1 ppm). The sensor was also tested on real samples, namely fresh and fermented juices and rice wine, by performing spike and recovery experiments. Once again, the SERS results on His detection were consistent with those obtained by means of HPLC measurements.

A colloidal SERS substrate of Au NPs was proposed in [83]. A detailed characterization of the substrate revealed a sensitivity in dihydrochloride His detection down to about 10 ppb (1 × 10^−7^ M).

Au NPs were also exploited to detect His in a work by Zhu, H. et al. [84]. An AuNPs substrate embedded in SiO_2_ was synthesized with the aim to achieve the clinical diagnosis of allergic diseases. The SERS measurements were performed both in water and serum, pointing out LODs values of 10^−8^ g/L (0.01 ppb) and 10^−6^ g/L (1 ppb), respectively. The exotic aspect is that the analysis of His SERS spectrum was performed on the low-frequency region, namely under 200 cm^−1^. Indeed, for an allergic sample a His mode at 83 cm^−1^ is possibly observed.

An alternative silica embedded metallic system is proposed in Ref. [85]. The article illustrates a detailed study on the optimization of His detection by means of SiO_2_@Au@Ag NPs as a function of solvent pH, incubation time of His in the colloidal NPs, target volume and material concentration. In the optimized condition a LOD of 3.7 ppm is reached.

An indirect SERS-tag strategy, specific for His detection, is described by Chen C. et al. [86]. The substrate is made up of magnetic Fe_3_O_4_@Au-aptamer nanoparticles that represent the histamine recognize probe and of a complementary DNA (c-DNA) carrying an Ag@4-MBN@Ag system which is the SERS signal probe. After the incubation of the two parts for a specific time, a core-satellite SERS aptasensor (Ag@4-MBN@Ag-c-DNA/Fe_3_O_4_@Au-aptamer) is obtained. Under a magnetic field, this SERS aptasensor exhibits a strong SERS signal characteristic of 4-MBN. As the content of His increases and it is bound by the aptamer, the 4-MBN signal decreases since the His-aptamer bound leads to the detachment of the SERS signal probe (containing 4-MBN). With this novel strategy a LOD of 0.65 × 10^−3^ ng/mL (0.65 × 10^−3^ ppb) was achieved. The tested samples were obtained by firstly incubating the sensor with His solution at different concentrations. Then, the obtained product was collected by magnetically separation, washed with a PBS buffer and redispersed into a PBS buffer solution. The SERS measurements were performed on the final samples dropped on silica substrate. This procedure was also used to perform measurements on spiked beer, obtaining good recovery values. Finally, the sensitivity of the sensor was tested on real samples, i.e., shoyu, rice vinegar, kirschwasser and fish samples. The detected His levels were compared with HPCL results and no significant differences were observed.

Finally, a recent study introduces a metal complex-based SERS nanoprobe made up of nitrilotriacetic acid-Ni^2+^ (NTA-Ni^2+^) and self-assembled Au NPs active substrates [87]. The substrate affinity is imparted by the NTA-Ni^2+^ which is able to capture and bring His close to Au NPs substrates. The sensitivity of this SERS substrate was tested with spiked human serum and a LOD of 1 μmol/L (≈0.1 ppm) was found.

## 6. SERS Detection of Sudan in Saffron and Other Spices

Sudan I–IV are a class of azo dyes, which are fat-soluble compounds mostly used as plastic colorants. The detection of saffron (Crocus Sativus) adulteration with Sudan is an interesting subject for the scientific community as Sudan dyes are forbidden in food trading [88] by the authorities of many countries including the European Union [89], because they can be dangerous for human health. The EU has set MRL for Sudan dyes in food at 0.5 ppm (Commission Directive 2006/33/EC). Any foods or food ingredients found to contain more than the established limit should be withdrawn from the market in the EU, and any foods containing the synthetic dyes Sudan I-IV, then, may not be placed on the market [90].

Besides saffron, Sudan is used as an adulterant in other powder spices such as chili powder, curry and paprika [91]. Typically, Sudan dyes as adulterants are in the low ppm concentration range [92].

**Figure 9:**
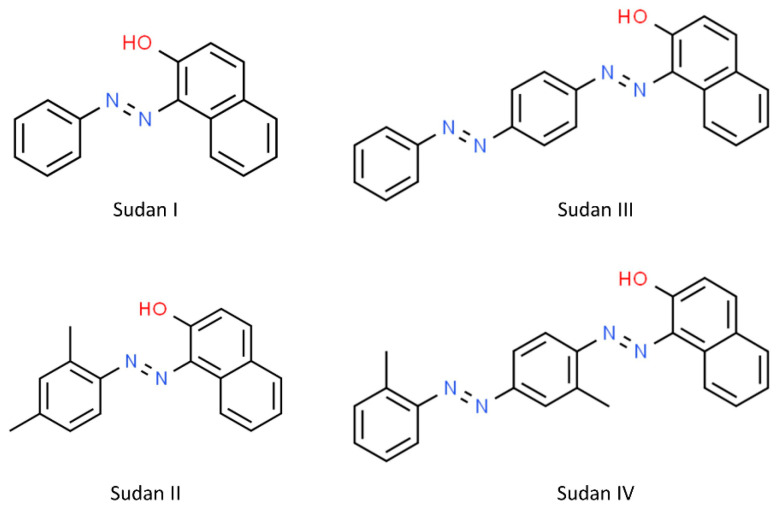
Molecular structures of Sudan I-IV, retrieved from [93].

Sudan dyes I-IV have similar chemical structures. They are aromatic compounds containing an azo group (-N=N-). The structures of Sudan I-IV are illustrated in Figure 9.

In a study by Y. Ou et al. [94], SERS experiments were carried out using Au–Ag core–shell colloidal nanospheres (Au@Ag) as a substrate. The method was applied in the analysis of standard solutions of Sudan I–IV and Sudan dyes extracted from spiked chili peppers. The LOD for Sudan I and II were 0.10 ppm, for Sudan III was 0.08 ppm, and for Sudan IV was 0.2 ppm. In chili flakes, the LDC was 1 ppm for Sudan I, II, and III and 2 ppm for Sudan IV. These figures are approximately ten times larger than those for standard solutions due to the interference of non-target molecules in the samples.

Detection of methanol extracted hydrophobic Sudan III in real food with sensitivity up to 9 mmol/L (≈3 × 10^3^ ppm) was accomplished by using hydrophobic surface modified silver nanoparticles (lipophilic sensor layers) [95]. In this work, the combination of SERS with microfluidics [96] significantly reduced the low-reproducibility problem by allowing high data acquisition rates and, consequently, enhanced statistical stability of the dataset.

**Figure 10:**
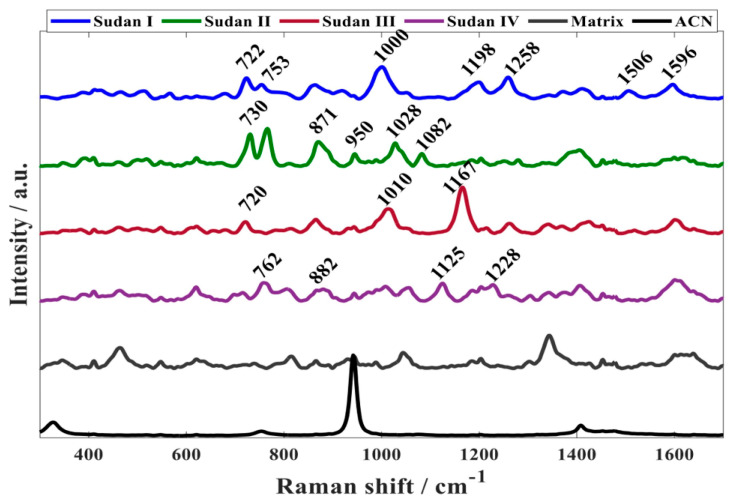
Representative SERS spectra of Sudan I, II, III, and IV in the concentrations ranging between 25 and 50 mmol/L (roughly 7.5 and 15 × 10^3^ ppm), the aggregated citrate-capped gold nanoparticles, and the acetonitrile solvent (ACN) (retrieved from [93]).

The ability of SERS to quantify Sudan I-IV dyes was investigated in a recent study by Alomar et al. [93]. SERS spectra were acquired using a benchtop Raman instrument and gold nanoparticles were employed as the SERS substrate. The authors demonstrated the feasibility of rapidly and simultaneously quantifying all four Sudan dyes in mixtures containing multiple Sudan dyes at different concentrations, with limits of detection around 10^−6^ mol/L (≈0.4 ppm). Stock solutions of the Sudan dyes were diluted in a mixture of water and Acetonitrile (1:10 *v*/*v*). Principal component analysis on the SERS spectra made it possible to distinguish and classify the Sudan dyes. Sudan II, III and IV are structurally derived from Sudan I (1-phenyl-azo-2-naphthol). Nonetheless, the SERS spectra acquired from the different Sudan dyes are markedly different (Figure 10). This is possibly due to the association of different parts of these molecules with the gold nanoparticles. The interaction between the negatively charged surface of the citrate-capped nanoparticles and the diazene functional group of the sample molecules (the -N=N- can become positively charged) is likely to play an important role. Moreover, ordinary Raman spectra of Sudan dyes (see, e.g., Ref. [94]) are strongly modified by the interaction with the SERS substrate. This last effect is ubiquitous in Raman and SERS spectroscopy.

**Figure 11:**
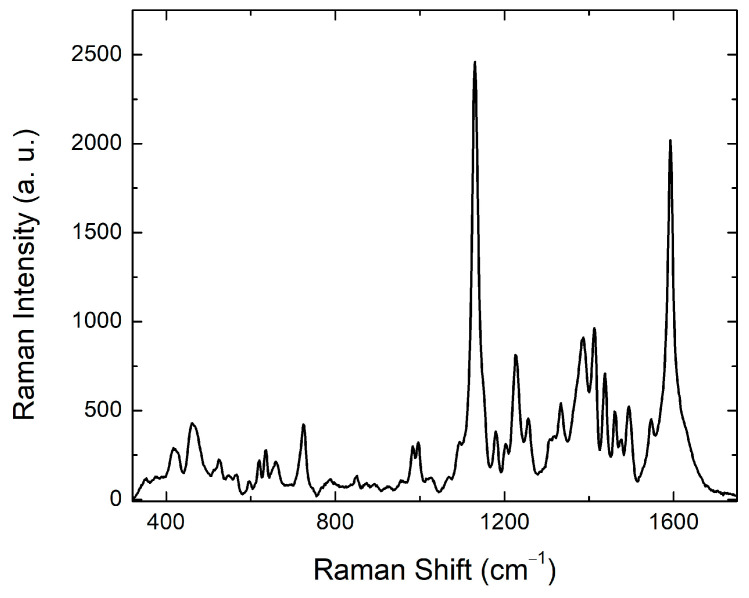
SERS spectrum of 0.2 ppb Sudan III collected on an Ag-nanoplates chip with laser wavelength λ = 638 nm.

Preliminary SERS investigations on Sudan dyes have recently been performed in our laboratory. Figure 11 reports the SERS spectrum of 5 × 10^−7^ mol/L (≈0.2 ppb) Sudan III collected by means of a Horiba Xplora Raman microspectrometer (laser wavelength = 638 nm). The substrate is an Ag-nanoplates chip. The measurements were performed on solid chips immersed in a solution of Sudan dissolved in acetonitrile, and then dried.

Two prominent lines are observed at about 1130 and 1590 cm^−1^. According to the mode assignments reported in [94] the line at 1130 cm^−1^ can be assigned to C-H in-plane bending, O-H in-plane bending, C-H scissoring in benzene rings, and C-H stretching, whereas the line at 1590 cm^−1^ can be assigned to C-C scissoring in benzene rings, and N=N stretching.

## 7. Concluding Remarks

Food safety monitoring is nowadays an unquestionable issue for producers, consumers, public welfare and private and public stakeholders. The need for prompt, easy-to-use and accurate methodologies and techniques is thus increasing. Among different approaches, optical probes are surely suitable. In particular, it is highly desirable to have optical probes able to selectively detect substances using molecular fingerprints with sensitivity down to ppm or, better, ppb. In this frame, in recent decades, SERS has appeared to be a unique tool for food safety, matching the molecular selectivity typical of Raman spectroscopy, with higher sensitivity due to plasmonic-assisted field enhancement and the possibility of in situ operation. In this review, we tried to highlight the SERS potential for monitoring different food safety issues, namely pesticides in rice cultivations (glyphosate), antibiotics (tetracycline) in soils from sludge dispersion, intolerance agents (histamine) in wine and adulterants (Sudan) in saffron and other spices. Our selected focus was dictated by a geographic interest, as all the above-mentioned targets are extremely relevant for agriculture in the Oltrepò area. Table 1 shows the state of the art concerning the monitoring of the analytes targeted in this work. This summary provides evidence of LODs for each analyte in relation to the experimental conditions.

The plethora of research mentioned in the present review indicates that SERS is able to detect and discriminate between different analytes with sensitivity in compliance with EU food safety rules and laws. Nevertheless, further progress must be made so that SERS can be recognized as a routine technique and can be included in standard protocols to ensure food purity and integrity. This step strongly depends on the advancement in the targeted tailoring of nanostructured substrates, where it is crucial to define the architecture of the SERS tag and its functionalization. These features can in turn depend on the measurement conditions, especially when on-site operation is required. We also point out that the SERS approach can become a solid benchmark in terms of food safety assessment. This should benefit from the possibility of processing a large amount of data, aimed at strengthening the robustness of analytics. This is necessary because of the intrinsic variability of SERS signals. For this purpose, a very important help can be derived from the application of deep learning strategies to SERS data analyses.

## Figures and Tables

**Table 1:** Essential information about the SERS works presented in this review.

Analyte	Instrument	Substrate	Mechanism	Sensitivity (LOD)	References
Glyphosate	b ^1^, 532 nm	modified Ag NPs ^3^	direct	170 ppm	[38]
	p ^2^, 785 nm	Au NPs	direct	17 ppm	[38]
	b, 633 nm	Ag NPs	direct	not available	[39,41,42]
	b, not available	modified Au NPs	indirect	0.0034 ppb	[43]
	b, 633 nm	Ag NPs	direct	3.2 ppm	[44]
	b, 633 nm	Ag NPs	direct	1.3 ppm	[45]
	b, 780 nm	Ag NPs	direct	0.0016 ppb	[46]
	b, 647 nm	Ag NPs	direct	40 ppb	[47]
	b, 633 nm	Ag NPs	direct	0.0085 ppb	[48]
	b, 638 nm	Ag NPs	direct	110 ppb	[49]
	b, 785 nm	Ag NPs	direct	3.14 ppb	[50]
	b, 532 nm	TiO_2_/Ag heterostr.	direct	0.1 ppb	[51]
	p, not available	Au-Ag nanochains	indirect	5 ppb	[52]
	p, 785 nm	Au NPs	direct	1.3 ppb	[53]
Tetracycline	b, 633 nm	Ag NPs	direct	0.1 ppm	[64]
	p, 785 nm	Ag NPs	direct	0.01 ppm	[65]
	b, 785 nm	Ag NPs	direct	0.44 ppb	[61]
	b, 532 nm	Ag NPs	direct	0.28 ppb	[66]
	b, 532 nm	Ag NPs	direct	0.44 ppb	[68]
Histamine	b, 532 nm	modified Ag NPs	direct	10^−4^ ppb	[80]
	b 785 nm	MIPs ^4^-Ag NPs	direct	0.04 ppm	[81]
	b, 785 nm	carbon dots/Au NPs	direct	1 ppm	[82]
	b, 785 nm	Au NPs	direct	10 ppb	[83]
	b, 514.5 nm	Au NPs	direct	0.01 ppb	[84]
	b, 532 nm	SiO_2_@Au@Ag NPs	direct	3.7 ppm	[85]
	b, 532 nm	modified Ag NPs	indirect	0.65 10^−3^ ppb	[86]
	b, 633 nm	Au NPs	indirect	0.1 ppm	[87]
Sudan I, II, III, IV	b, 785 nm	Au NPs	direct	0.4 ppm	[93]
Sudan I, II, III, IV	b, 633 nm	Au@Ag NPs	direct	0.08 to 0.2 ppm	[94]
Sudan III	b, 488 nm	modified Ag NPs	direct	3 × 10^3^ ppm	[95]
Sudan III	b, 638 nm	Ag NPs	direct	LOD < 0.2 ppb	original results

^1^ benchtop instrument; ^2^ portable instrument; ^3^ NanoParticles; ^4^ Molecularly Imprinted Polymers.

## Data Availability

Not applicable.
